# Glutamate Deregulation in Ketamine-Induced Psychosis—A Potential Role of PSD95, NMDA Receptor and PMCA Interaction

**DOI:** 10.3389/fncel.2017.00181

**Published:** 2017-06-28

**Authors:** Malwina Lisek, Bozena Ferenc, Maciej Studzian, Lukasz Pulaski, Feng Guo, Ludmila Zylinska, Tomasz Boczek

**Affiliations:** ^1^Department of Molecular Neurochemistry, Faculty of Health Sciences, Medical UniversityLodz, Poland; ^2^Department of Molecular Biophysics, Faculty of Biology and Environmental Protection, University of LodzLodz, Poland; ^3^Laboratory of Transcriptional Regulation, Institute of Medical BiologyLodz, Poland; ^4^Department of Pharmaceutical Toxicology, School of Pharmacy, China Medical UniversityShenyang, China; ^5^Boston Children’s Hospital and Harvard Medical SchoolBoston, MA, United States

**Keywords:** calcium homeostasis, ketamine, NMDA receptor, plasma membrane Ca^2+^-ATPase, PSD95, psychosis

## Abstract

Ketamine causes psychotic episodes and is often used as pharmacological model of psychotic-like behavior in animals. There is increasing evidence that molecular mechanism of its action is more complicated than just N-methyl-D-aspartic acid (NMDA) receptor antagonism and involves interaction with the components of calcium homeostatic machinery, in particular plasma membrane calcium pump (PMCA). Therefore, in this study we aimed to characterize brain region-specific effects of ketamine on PMCA activity, interaction with NMDA receptor through postsynaptic density protein 95 (PSD95) scaffolding proteins and glutamate release from nerve endings. In our study, ketamine induced behavioral changes in healthy male rats consistent with psychotic effects. In the same animals, we were able to demonstrate significant inhibition of plasma membrane calcium ATPase (PMCA) activity in cerebellum, hippocampus and striatum. The expression level and isoform composition of PMCAs were also affected in some of these brain compartments, with possible compensatory effects of PMCA1 substituting for decreased expression of PMCA3. Expression of the PDZ domain-containing scaffold protein PSD95 was induced and its association with PMCA4 was higher in most brain compartments upon ketamine treatment. Moreover, increased PSD95/NMDA receptor direct interaction was also reported, strongly suggesting the formation of multiprotein complexes potentially mediating the effect of ketamine on calcium signaling. We further support this molecular mechanism by showing brain region-specific changes in PSD95/PMCA4 spatial colocalization. We also show that ketamine significantly increases synaptic glutamate release in cortex and striatum (without affecting total tissue glutamate content), inducing the expression of vesicular glutamate transporters and decreasing the expression of membrane glutamate reuptake pump excitatory amino acid transporters 2 (EAAT2). Thus, ketamine-mediated PMCA inhibition, by decreasing total Ca^2+^ clearing potency, may locally raise cytosolic Ca^2+^ promoting excessive glutamate release. Regional alterations in glutamate secretion can be further driven by PSD95-mediated spatial recruitment of signaling complexes including glutamate receptors and calcium pumps, representing a novel mechanism of psychogenic action of ketamine.

## Introduction

Ketamine, a high affinity non-competitive antagonist of N-methyl-D-aspartic acid receptor (NMDAR) is known to induce schizophrenia-like symptoms (Becker et al., 2003). Growing body of evidence suggests a blockage of NMDAR channel pore by ketamine as an underlying mechanism for drug-induced psychosis observed both in humans and animal models (Keilhoff et al., 2004; Tomiya et al., 2006). Pharmacologically controlled induction of psychotic-like behavior is now widely used to mimic the symptoms and molecular abnormalities of this disease in various animal models (Chatterjee et al., 2012; Koh et al., 2016). Schizophrenic individuals often demonstrate impaired dopaminergic and GABAergic neurotransmission which was shown in neurochemical and neurophysiological studies to be caused by NMDA receptor dysfunction (Krystal et al., 1994; Javitt, 2007). Furthermore, NMDA receptors are located on brain circuits that regulate dopamine release. Thus, dopaminergic deficits may be secondary to underlying glutamatergic dysfunction, mainly in striatal and prefrontal brain regions (Javitt, 2010; Sleigh et al., 2014), although it is now more evident that ketamine acts on multiple brain areas and influences the function of components other than NMDAR (Lindefors et al., 1997; Howes and Kapur, 2009; Koh et al., 2016).

One of the critical cellular function affected by ketamine is Ca^2+^-dependent signaling, which can mediate at least some of the psychotomimetic effects of ketamine (Lidow, 2003; Bojarski et al., 2010). It is because cytosolic Ca^2+^ is a universal signaling messenger and disruption of the highly interdependent calcium-dependent enzymes leads to the propagation of aberrant signaling with long-lasting consequences on neuronal functioning. The tight control of cytosolic calcium ([Ca^2+^]_c_) rises and fast termination of Ca^2+^ signal are necessary to determine neurotransmitter release and synaptic communication, both controlling multiple vital functions. It has been reported that ketamine may, at least temporarily, increase the number and function of synaptic connections by promoting glutamate burst (Sleigh et al., 2014).

The critical role in restoration of neuronal resting [Ca^2+^]_c_ following even subtle changes in Ca^2+^ concentration is ascribed to the plasma membrane calcium ATPase (PMCA). In the brain, this enzyme is represented by four main isoforms with several splice variants (Lopreiato et al., 2014; Strehler, 2015). The neural tissue is especially rich in PMCA2 and PMCA3 isoforms; their location is specific to excitable cells, whereas PMCA1 and PMCA4 are widely expressed throughout the body. Location and function of PMCAs at the pre- and post-synaptic sites is regulated, among others, by their molecular interaction with postsynaptic-density-95/discs large/zona occludens-1 domain (PDZ-domain)-containing proteins (Kim et al., 1998; DeMarco and Strehler, 2001; Kruger et al., 2010). This class of proteins is found in specific subcellular structures and plays a central role in the organization of biologically active complexes in the plasma membranes. Up to now, more than 4000 different interactions between synaptic proteins and cellular PDZ proteins have been identified (Cui et al., 2007; Feng and Zhang, 2009). Postsynaptic density protein 95 (PSD95) is one of the most ubiquitous scaffolding proteins containing three PDZ domains and it is concentrated at glutamatergic synapses (Elias et al., 2008). PSD95 plays a fundamental role in clustering of transmembrane proteins within specific subcellular domains, thus modifying their functionality. It has been shown that PMCA2b and PMCA4b interact with PSD95 via the PDZ domain-binding motif and this interaction is thought to tether PMCA to specialized calcium microdomains, the centers of integration of membrane signaling (DeMarco and Strehler, 2001; Kruger et al., 2010). More importantly, PMCA/PSD95 complex also recruits the NMDA type glutamate receptor subunits, NMDAR1 and NMDAR2A (Garside et al., 2009). This raises the possibility of formation of multiprotein complexes functionally coupling NMDAR and PMCA. Such interaction would allow rapid response to local Ca^2+^ increases due to bringing PMCA in close proximity to Ca^2+^ entry sites. As Ca^2+^ is critical for neurotransmitter release, recruitment of NMDAR/PMCA into presynaptic active zones may modulate the amplitude of Ca^2+^ rises, thus affecting synaptic transmission (Blaustein et al., 2002). The PSD95-mediated clustering of PMCA and NMDAR at postsynaptic membrane may also affect Ca^2+^ signaling, modulate the recruitment of signaling molecules and influence the secretion of neurotransmitter through retrograde signaling (Fitzsimonds and Poo, 1998). Despite that, the functional consequences of PSD95 clustering are not well known and the alterations in PMCA/PSD95 or NMDAR/PSD95 complex formation have not been linked to psychogenic effects of ketamine.

Based on our previous findings (Boczek et al., 2015b), we hypothesize that ketamine may affect PMCA membrane content and inhibit pump activity in different brain areas. One predictable effect of such action would be the deregulation of neuronal Ca^2+^ homeostasis, putatively triggering regionally enhanced glutamate neurotransmission and/or excitotoxicity. However, as NMDAR and PMCA are known to be coupled by PSD95 scaffolding protein, we also expect to observe ketamine-mediated alterations in NMDAR/PSD95/PMCA complex formation which may represent a compensatory mechanism to counterbalance local Ca^2+^ and glutamate elevations. Therefore, in this study we aimed to characterize brain region-specific effects of chronic ketamine administration (30 mg/kg) on PMCA activity and NMDA/PMCA clustering through PSD95 protein and to draw conclusions on the role of PMCA in glutamate overstimulation which is thought to be pivotal for ketamine-evoked psychosis.

## Materials and Methods

### Reagents

All reagents including mouse monoclonal anti-PSD95 antibody (Cat. No. P246), if not separately mentioned, were purchased from Sigma-Aldrich (Germany). Ketamine (in the form of ketamine hydrochloride) was from Biowet (Poland). Mouse monoclonal anti-GAPDH (Cat. No. sc-32233) or rabbit polyclonal anti-Na^+^/K^+^-ATPase α antibodies (Cat. No. sc-28800), Protein A/G PLUS-Agarose and BCIP/NBT were from Santa Cruz Biotech (USA). Protein Assay Kit was from Bio-Rad (USA). Rabbit polyclonal anti-PMCA1 (Cat. No. PA1-914), rabbit polyclonal anti-PMCA2 (Cat. No. PA1-915), rabbit polyclonal anti-PMCA3 (Cat. No. PA1-916), mouse monoclonal anti-PMCA4 (Cat. No. MA1-914), rabbit polyclonal anti-NMDAR1 (Cat. No. PA3-102), rabbit polyclonal anti-NMDAR2A (Cat. No. A-6473), rabbit polyclonal anti-NMDAR2B (Cat. No. PA3-104), mouse monoclonal 5F10 (Cat. No. MA3-914), rabbit polyclonal anti-VGLUT1 (Cat. No. 48-2400), normal mouse IgG1, normal rabbit IgG as well as secondary antibodies conjugated with Alexa Fluor 488 and Alexa Fluor 568 were purchased from Thermo Scientific (USA). Rabbit monoclonal anti-PSD95 antibody (Cat. No. ab76115) used for immunohistochemical staining, rabbit polyclonal anti-VGLUT2 (Cat. No. ab84103) and rabbit polyclonal anti-excitatory amino acid transporters 2 (EAAT2) (Cat. No. ab41621) were from Abcam (UK). γ^32^-ATP (500 Ci/mmol) was from Perkin-Elmer (USA). Protein Assay Kit was from Bio-Rad (USA). Primers were synthesized in the Institute of Biochemistry and Biophysics (Poland).

### Animals

Male Wistar rats (10–12 week-old, 250–275 g) obtained from an in-house animal facility of Medical University of Lodz were group-housed at four per cage at room temperature (RT; 23 ± 2°C) and 12/12 h light/dark (8.00 a.m. to 8.00 p.m.) cycle. Food pellets and water were provided *ad libitum*. Males were used to avoid estrus cycle-associated hormonal changes in female rats that could have confounded results. Randomly chosen rats were injected with ketamine (30 mg/kg, i.p.) once daily for FIVE consecutive days while the control group received saline only (0.5 ml/kg). Dose, route of administration and schedule were in accordance with previous studies showing psychotic-like alterations following 5-day ketamine treatment (Becker et al., 2003; Keilhoff et al., 2004; Boczek et al., 2015a). Animals were killed by decapitation immediately after behavioral testing and no longer than 3 h after final ketamine injection. Brains were immediately removed and placed on ice-chilled petri dish. The cortex, cerebellum, hippocampus and striatum were dissected, quickly frozen with liquid nitrogen and stored at −80°C until use. All experiments were conducted in accordance with the European Communities Council Directive of 24 November 1986 (86/609/EEC) and were in compliance with *the Association for Assessment of Laboratory Animal Care* guidelines for animal use. The Institutional Animal Care and Use Committee at Medical University of Lodz approved the study. To minimize group size and animal suffering, the same animals were used for determination of PMCA hydrolytic and phosphotransferase activity and the tissue lysates obtained from the same animals were used for western blot and co-immunoprecipitation assays.

### Open-Field Test

The open field test was performed essentially as described by Colaianna et al. (2010). Approximately 30 min after final ketamine injection animals (*n* = 16) were placed into dark plastic rectangular arena (40 × 30 × 40 cm) and were allowed to acclimatize for 10 min. Motor activity was measured for 20 min and the scoring was performed using an automated software (ANY-maze tracking system, Stoelting, Kiel, WI, USA). The following behavioral parameters were assessed: immobility time; total grooming activity consisting of face grooming, head washing and body grooming; number of square crossings with both forepaws; rearing (forequarters raised, body inclined vertically) and total sniffing. Since in the open-field test, the avoidance of open space can reflect anxiety behavior, the number of entries into two centrally located squares was also assessed. The arena was cleaned with 70% ethanol after each animal. All measurements were performed between 10 a.m. and 3 p.m. by an investigator who was blind to the animal experimental group. The corresponding control group (*n* = 16) receiving saline only was also tested under identical experimental conditions.

### Synaptosomal Preparation

Cerebellar, hippocampal and striatal synaptosomes from ketamine- and saline-treated rat brains were obtained based on the method of Gray and Whittaker (1962). Briefly, selected brain parts were ice-cold homogenized using 10 strokes of a teflon-glass homogenizer in a buffer containing 0.32 M sucrose, 5.0 mM HEPES, pH 7.5, 0.1 M EDTA, 5 mM dithiothreitol supplemented with protein inhibitor cocktail. Homogenates were first centrifuged for 20 min, 1000× *g*, at 4°C and the supernatant was next centrifuged for 30 min, 20,000× *g*, at 4°C. The pellet enriched in crude synaptosomes were collected, suspended in 2 ml of the above buffer, layered onto a sucrose gradient (0.8 M: 1.2 *M* = 1:1) and centrifuged at 100,000× *g* for 1 h. Purified synaptosomes were suspended in an ice-cold artificial cerebrospinal fluid (aCSF) containing 132 mM NaCl, 3 mM KCl, 2 mM CaCl_2_, 2 mM MgCl_2_, 10 mM HEPES, pH 7.4, 10 mM glucose, 1.2 mM NaH_2_PO_4_ and cocktail of protease inhibitors and were kept at 4°C for immediate use. When used for glutamate release experiments, synaptosomes were also resuspended in calcium-free aCSF.

### Measurement of Ca^2+^-ATPase Hydrolytic and Phosphotransferase Activity

The Mg^2+^, Ca^2+^-ATPase in synaptosomes was determined according to the method of Lin and Morales (1977). In brief, ~10 mg of synaptosomes were pre-incubated for 2 min at 37°C in a buffer containing 50 mM Tris-HCl, pH 7.4, 100 mM KCl, 3 mM MgCl_2_, 1 mM EGTA, 0.1 mM ouabain and 1.024 mM CaCl_2_ (10 μM Ca^2+^ free) in a total volume of 200 μl. The PMCA activity measured in the presence of Ca^2+^ but in the absence of CaM is referred to as basal activity and that in the presence of Ca^2+^ and 70 nM CaM as CaM-stimulated activity. The reaction was initiated by the addition of 3 mM ATP and the activity was measured following 1 min incubation at 37°C. The absorbance of the colored complex formed by P_i_ and molybdovanadate was read at 350 nm and the amount of Pi was determined by a calibration curve using K_2_HPO_4_ solutions as standards. The activity was expressed as μmoles of P_i_/mg protein/h.

Formation of phosphoenzyme intermediate reflecting Ca^2+^-ATPase phosphotransferase activity was measured following the procedure described by Guerini et al. (1999) with some modifications. Synaptosomes (~1 mg) were resuspended in a buffer A containing 25 mM Tris/HCl, pH 6.8, 100 mM KCl, 100 μM Ca^2+^ and kept on ice. The reaction was started by the addition of 0.3 μM γ^32^-ATP (500 Ci/mmol) and stopped 60 s later with 7% trichloroacetic acid. Samples were centrifuged at 10,000× *g* for 10 min, the pellet was washed, resuspended in a buffer B (50 mM Tris/HCl, pH 6.3, 150 mM NaCl, 1 mM EDTA, 0.1% Tween-20, 0.5% SDS), kept on ice for 10 min and then centrifuged at 10,000× *g* for 30 min. ~5 μg of 5F10 antibodies or antibodies specific to PMCA1, PMCA2, PMCA3 or PMCA4 were added to supernatant aliquots and the samples were incubated overnight at 4°C followed by an addition of 20 μl of sepharose-conjugated A/G protein beads. After 4 h of incubation at 4°C, beads were spun down at 1000× *g* for 5 min and the pellet was washed three times with buffer B, resuspended in buffer C (70 mM Tris/HCl, pH 6.4, 5% SDS, 5% dithiothreitol, 8 M urea), incubated at RT for 20 min, separated on acidic gels and exposed to X-ray films (4°C, 3–5 days). The films were scanned and quantified densitometically with ImageJ, version 1.49 (NIH, USA). The results were expressed as OD/mg protein.

### Preparation of Tissue and Synaptosomal Lysates

The lysates were obtained as described by Harlow and Lane (1988). Selected brain parts (~100 mg pieces) were homogenized at 4°C in five volumes of RIPA buffer supplemented with 1 mM PMSF, 2 mM Na_3_VO_4_ and protease inhibitor cocktail with 10–15 strokes of tight-fitting Dounce homogenizer. Homogenates were left for 30 min on ice, sonicated 3 × 5 s cycles using 100 Ultrasonic Cell Disrupter (Virtis Virsonic, USA) and centrifuged at 17,000× *g* for 20 min. The supernatants referred to as tissue lysates were collected. Synaptosomal pellet obtained as described in *Synaptosomal preparation* section was lysed with RIPA buffer followed by sonication and centrifugation to get synaptosomal protein lysate. Protein concentration was measured using Bio-Rad Protein Assay Kit.

### Western Blotting

40–60 μg of tissue or synaptosomal lysate proteins were separated using either 8% or 10% SDS-PAGE and transferred onto nitrocellulose membrane (Boczek et al., 2014). The membranes were first blocked with 6% BSA in TBS-T (10 mM Tris-HCl, pH 7.4, 150 mM NaCl, 0.05% Twwen-20) for 2 h at RT. Next, the membranes were incubated with primary antibodies at 4°C for 12 h. The final dilutions for primary antibodies were as follows: 5F10 (1:1000), anti-PMCA1 (1:1000), anti-PMCA2 (1:750), anti-PMCA3 (1:750), anti-PMCA4 (1:1000), anti-PSD95 (1:2000), anti-NMDAR1 (1:1000), anti-NMDAR2A (1:1000), anti-NMDAR2A (1:1000), anti-VGLUT1 (1:1000), anti-VGLUT2 (1:1500), anti-EAAT2 (1:1500), anti-GAPDH (1:1000) and anti-Na^+^/K^+^ ATPase (1:1500). Membranes were washed three times with TBS-T and incubated at RT for 4 h with secondary antibodies (1:5000) conjugated to alkaline phosphatase. Bands were visualized with BCIP/NBT solution according to the manufacturer’s protocol. Membranes were scanned and the intensity of bands was quantified densitometrically using ImageJ, version 1.49 (NIH, Bethesda, MD, USA). The results were expressed as arbitrary units (AU) after normalization to Na^+^/K^+^- ATPase protein.

### Co-Immunoprecipitation

For co-immunoprecipitation assay, 800 μg of brain tissue lysate proteins was pre-cleared with 20 μl of Protein A/G PLUS-Agarose beads for 2 h at 4°C and centrifuged at 14,000× *g* for 5 min. The pre-cleared supernatant was incubated with anti-PSD95 antibodies (~6 μg of antibodies/800 μg of lysate proteins) for 2 h at 4°C followed by an incubation with 25 μl of Protein A/G PLUS-Agarose beads at 4°C overnight. Normal mouse IgG at the same concentration was used as a negative control. The immunocomplexes were recovered by centrifugation at 14,000× *g* for 5 min, washed three times with PBS, eluted with 30 μl of SDS-PAGE sample buffer (62.5 mM Tris-HCl, pH 6.8, 10% glycerol, 2% SDS, and 0.001% bromphenol blue) containing 5% β-mercaptoethanol and subjected to immunoblotting as described in *Western blotting* section. The membranes with immunoprecipitated PSD95 protein were probed with anti-NMDAR1, anti-NMDAR2A, anti-NMDAR2B, 5F10 (1:1000), anti-PMCA2 (1:1000) or anti-PMCA4 (1:1000) antibodies. Bands were visualized and scanned as described in *Western blotting* section. The results are presented as OD/mg protein.

### Immunohistochemistry and Confocal Imaging

Dissected cortex, cerebellum, hippocampus and striatum were washed 3 × 5 min with TBS and immediately fixed using 4% paraformaldehyde in an ice-cold PBS for 30 min. Following several washes with TBS, tissues were paraffin embedded and cut in 10-μm-thick sections at the Laboratory of Microscopic Imaging and Specialized Biological Techniques of Faculty of Biology and Environmental Protection (University of Lodz) using a microtome (Leica, Germany). The sections were mounted onto glass slides coated with poly-L-lysine and dried overnight at 37°C. Following deparaffinization and rehydration, tissue sections were subjected to antigen retrieval by microwaving in citrate buffer (pH 6.0) for 5 min, subsequent cooling down at RT for 3 min and reheating for 5 min. After washing with PBS, sections were sequentially incubated with 10% normal goat serum PBS solution for 1 h at RT, primary antibody solution or isotype control (day 1: anti-PMCA4, 1:100 or normal mouse IgG1, 1:100; day 2: anti-PSD95, 1:100 or normal rabbit IgG, 1:100) overnight at 4°C, washed with PBS (3 × 10 min, RT) and incubated with appropriate secondary antibody (Alexa Fluor 568 conjugated anti-mouse IgG1 or Alexa Fluor 488 conjugated anti-rabbit IgG, 1:1000) for 1 h at RT. Finally, nuclei were stained for 30 min with 5 μM Hoechst33342 and sections were embedded in self-made PVA-based mounting medium. Imaging was performed on a 780 LSM confocal microscope (Zeiss, Germany) equipped with Plan-Apochromat 63×/1.4 Oil DIC M27 objective. Images were acquired in 1 μm wide optical sections, in fields of view 150 μm by 150 μm (1960 × 1960 pixels). Scans were performed in three channels (excitation wavelengths 405 nm, 490 nm, 568 nm; emission wavelength ranges 410–509 nm, 498–570 nm, 572–653 nm, respectively) with a pixel dwell time of 7.32 μs. For each brain region (control or ketamine treated), images were taken in three different fields of view within the corresponding anatomical structures. Colocalization study was performed using ZEN2012 software (Zeiss, Germany). Since PMCA4 as a plasma membrane transporter localizes at specific subcellular sites, while PSD95 is more diffusible, we have calculated the percentage of PSD95-positive pixels overlapping with PMCA4-positive pixels for each brain region and used it to describe PSD95/PMCA4 colocalization. For this purpose, overlap of PSD95 fluorescence signal with PMCA4 signal was calculated for regions of interest selected across each field of view by dividing the number of pixels positive (exceeding a threshold) for signals in both channels (PMCA4-positive sites which are also PSD95-positive) by the total number of pixels positive for PMCA4 signal. Appropriate isotype controls were performed. None of the immunofluorescence reactions revealed unspecific fluorescent signal in the negative controls.

### RNA Isolation and Real-Time PCR

Total brain RNA was isolated using Trizol reagent according to the manufacturer’s protocol. The oligo (dT) primers and 1 μg of purified RNA were then used for cDNA synthesis in a reaction catalyzed by M-MLV reverse transcriptase. The gene expression level was quantified in a real-time PCR reaction using Maxima SYBR Green Master Mix in the following conditions: 15 min at 95°C followed by 40 cycles at 95°C for 15 s, 60°C for 30 s and 72°C for 30 s using Abi Prism 7000 sequence detection system (Applied Biosciences). Following normalization to the expression of endogenous *Gapdh*, the fold change of each target gene was calculated using comparative 2^−∆∆Ct^ method (Livak and Schmittgen, 2001). Specificity of the primers was confirmed by running a melting curve after each reaction. The sequence of the primers was as follows: VGLUT1 (*Slc17a7*): 5′-GGCAGTTTCCAGGACCTCCACTC-3′ and 5′-GCAAGAGGCAGTTGAGAAGGAGAGA G-3′; VGLUT2 (*Slc17a6*): 5′-GGGTATTTGGTCTGTTTGGTG TCCTG-3′ and 5′-CAGCACAGCAAGGGTTATGGTCAC-3′; EAAT2 (*Slc1a2*): 5′-TAACTCTGGCGGCCAATGGAAAG T-3′ and 5′-ACGCTGGGGAGTTTATTCAAGAAT-3′; *Gapdh*: 5′-GGTTACCAGGGCTGCCTTC T-3′ and 5′-CTTCCCATTCTCAGCCTTGACT-3′. The sequence for *Slc17a7* and *Slc17a6* was designed by Doyle et al. (2010), for *Gapdh* by Sobczak et al. (2010) and for *Slc1a2* the primers were designed using GenScript Primer Design Tool (USA).

### Determination of Glutamate/Glutamine Concentration and Synaptosomal Glutamate Release

Intrasynaptosomal glutamate and glutamine content was measured with *Glutamine and Glutamate Determination Kit* according to manufacturer’s instructions (Sigma-Aldrich, Germany).

Glutamate release from synaptosomal preparations was measured by fluorometric assay essentially as described by Nicholls et al. (1987). Briefly, ~100 μl synaptosomes (~1 mg/ml) suspended in aCSF supplemented with 2 mM NADP^+^ and 6.32 U L-glutamic acid dehydrogenase (and 2 mM CaCl_2_ whenever appropriate) was distributed into each of the 96 wells. Synaptosomes were depolarized with 30 mM KCl 5 min thereafter and the increase in NADPH fluorescence was monitored over a 10 min time period. Fluorescence emission was recorded at 450 nm and the excitation wavelength was set at 360 nm. Each experimental condition was carried out in triplicate on the same plate using Victor X3 fluorometer (Perkin Elmer, USA). The amount of released glutamate was calculated based on calibration curves prepared in parallel and the enzyme lag (Nicholls et al., 1987) was accounted for when converting rates to nmol/mg protein/10 min. The results obtained in the presence of 2 mM CaCl_2_ reflect total glutamate release whereas in the absence of Ca^2+^ in the reaction mixture as Ca^2+^-independent glutamate release.

### Data Analysis

The data are shown as means ± SD of n separate experiments (*n* ≥ 3) with the exact *n* value given under each figure. The normal data distribution was assessed using D’Agostino-Pearson (for *n* = 16) or Kolmogorov-Smirnov (for *n* < 16) tests. The comparisons between ketamine- and saline-treated groups were done using Student’s *t*-test or Mann-Whitney U test depending whether data passed normality tests. Two-way ANOVA with Bonferroni *post hoc* test was used to analyze ketamine and CaM effect on PMCA activity. *P* values were calculated using STATISTICA 8.0 (StatSoft). *P* < 0.05 was considered as statistically significant.

## Results

### Ketamine-Induced Behavioral Abnormalities

**Figure 1:**
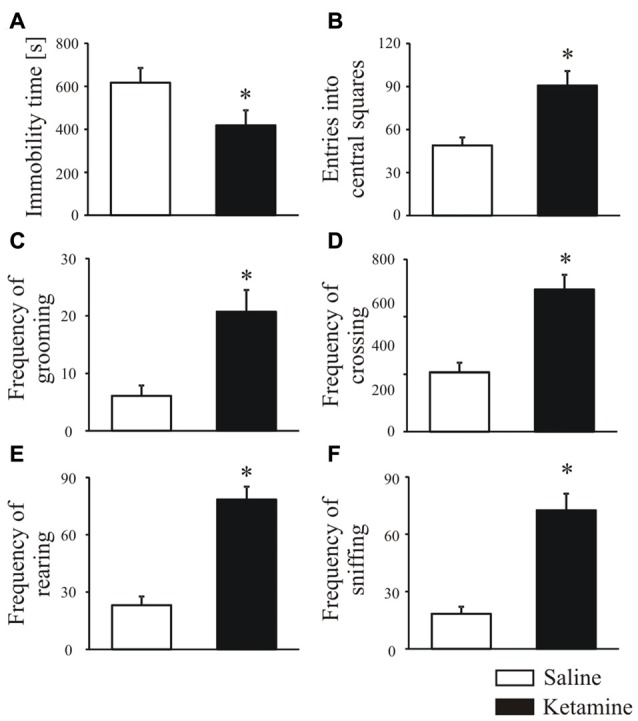
Animal behavior analysis in the open field test. Thirty minutes before behavioral testing, rats were intraperitoneally injected with last (fifth) ketamine dose (30 mg/kg). During the open field test, the total immobility time **(A)**, number of entries in the center area **(B)**, frequency of grooming **(C)**, crossing **(D)**, rearing **(E)** and sniffing **(F)** were recorded over 20 min and analyzed with ANY-maze tracking system. *n* = 16 for the control group (saline-treated); *n* = 16 for ketamine-treated group. *P < 0.05 ketamine treated vs. saline.

Rats were treated with ketamine (30 mg/kg, i.p.) once daily for five consecutive days, and an open-field test performed 30 min after last ketamine injection was used to score changes in animal behavior. Ketamine significantly induced hyperlocomotion reflected as shorter time of remaining immobile (Figure 1A, *P* < 0.0001, Mann-Whitney U test) and increased number of entries to the central squares suggesting a lower anxiety level (Figure 1B, *P* < 0.0001, Student’s *t*-test). In ketamine-treated group, the frequency of grooming (Figure 1C, *P* < 0.0001, Student’s *t*-test), crossing (Figure 1D, *P* < 0.0001, Mann-Whitney U test), rearing (Figure 1E, *P* < 0.0001, Student’s *t*-test) and sniffing (Figure 1F, *P* < 0.0001, Student’s *t*-test) was also significantly higher than that of control rats.

### Ketamine Effect on PMCA Activity in Rat Brain Regions

**Figure 2:**
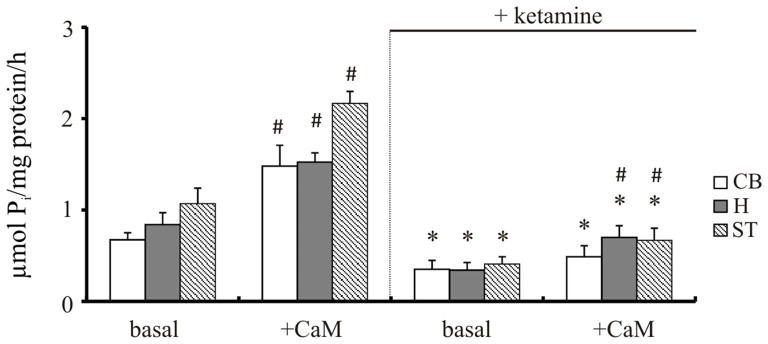
The hydrolytic activity of plasma membrane calcium pump (PMCA). The activity was measured in the presence or absence of 72 nM calmodulin (CaM) according to the method of Lin and Morales (1977) as described in “Materials and Methods” Section. *P < 0.05 ketamine treated vs. saline; ^#^*P* < 0.05 CaM-stimulated vs. basal activity, *n* = 5. CB, cerebellum; H, hippocampus; ST, striatum.

Our previous study demonstrated increased Ca^2+^ level in brain cells isolated from cortical regions following 5-day treatment with 30 mg/kg of ketamine (142 ± 25 nM vs. 116 ± 6 nM in control group; Lisek et al., 2016). The effect of ketamine is mainly attributable to the prefrontal cortex, although functional deficits in other brain regions such as hippocampus, cerebellum and striatum are also suggested to contribute to drug-induced psychotic symptoms (Lidow, 2003; Javitt, 2010; Sleigh et al., 2014). Therefore, we first compared the effect of ketamine treatment on PMCA activity in these brain regions using two independent methods—measurement of ATP hydrolysis and formation of phosphointermediate complex. Two way ANOVA revealed main effects of CaM (*F*_(1,16)_ = 54.96, *P* < 0.001; *F*_(1,16)_ = 106.3, *P* < 0.0001; *F*_(1,16)_ = 132.7, *P* < 0.0001), ketamine (*F*_(1,16)_ = 106.9, *P* < 0.001; *F*_(1,16)_ = 171.5, *P* < 0.0001; *F*_(1,16)_ = 335, *P* < 0.001) as well as CaM × ketamine interaction (*F*_(1,16)_ = 27.05, *P* < 0.001; *F*_(1,16)_ = 10.44, *P* = 0.0052; *F*_(1,16)_ = 50.62, *P* < 0.0001) on hydrolytic PMCA activity in cerebellum, hippocampus and striatum, respectively (Figure 2). CaM-dependent stimulation defined as a difference in activity in the presence of CaM vs. basal activity was markedly decreased in cerebellum (2.25 ± 0.64 vs. 1.41 ± 0.22, *P* = 0.025, Student’s *t* test) and striatum (2.05 ± 0.26 vs. 1.63 ± 0.1, *P* = 0.011, Student’s *t* test) following ketamine treatment. Similarly, ketamine decreased basal PMCA hydrolytic activity in cortical synaptosomes from 0.89 ± 0.21 to 0.47 ± 0.18 μmol/mg/h and reduced the stimulatory effect of CaM by 45% when compared to saline-treated control, as shown previously (Boczek et al., 2015b).

**Figure 3:**
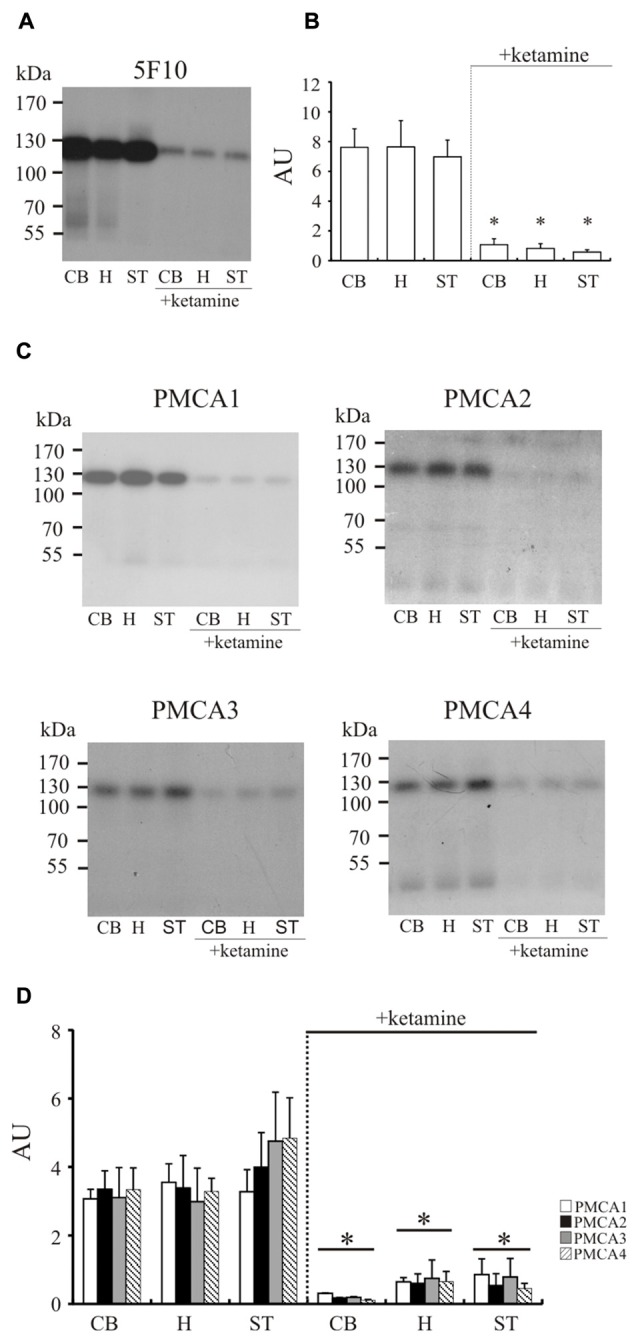
The measurement of phosphointermediate formation. Synaptosomal membranes were phosphorylated with 0.3 μM γ^32^-ATP, immunoprecipitated with 5F10 **(A)** or isoform-specific antibodies **(C)** and exposed to X-ray films for 3–5 days as described in “Materials and Methods” Section. The optical density (OD) of bands corresponding to the total amount of phosphoenzyme formed **(B)** or to particular PMCA phosphoisoform amount **(D)** was densitometically quantified. The results are presented as arbitrary units (AU) expressed as OD/mg protein. Representative autoradiograms are shown. *P < 0.05 ketamine treated vs. saline, *n* = 5. CB, cerebellum; H, hippocampus; ST, striatum.

To confirm these results, we immunoprecipitated ^32^P-phosphointermediate using 5F10 antibodies, as well as antibodies specific to particular PMCA isoforms (Figure 3). In line with the above data, we detected significantly lower autoradiographic signal in cerebellum (−85 ± 7%, *P* < 0.0001, Student’s *t*-test), hippocampus (−88 ± 5%, *P* < 0.0001, Student’s *t*-test) and striatum (−91 ± 3%, *P* < 0.0001, Student’s *t*-test). Similar level of inhibition was previously observed by us in cortex (Boczek et al., 2015b), and all these results reflect compromised total phosphotransferase activity of PMCA in response to chronic ketamine administration. Comparable magnitude of signal loss was observed when particular PMCA isoforms were immunoprecipitated in the same way (Figure 3). This suggests that each PMCA isoform is similarly vulnerable to inhibition by ketamine and the observed diminished formation of phosphointermediate may be a result of decreased activity of all PMCAs.

### Ketamine Effect on PMCA Amount in Synaptosomal Membranes

**Figure 4:**
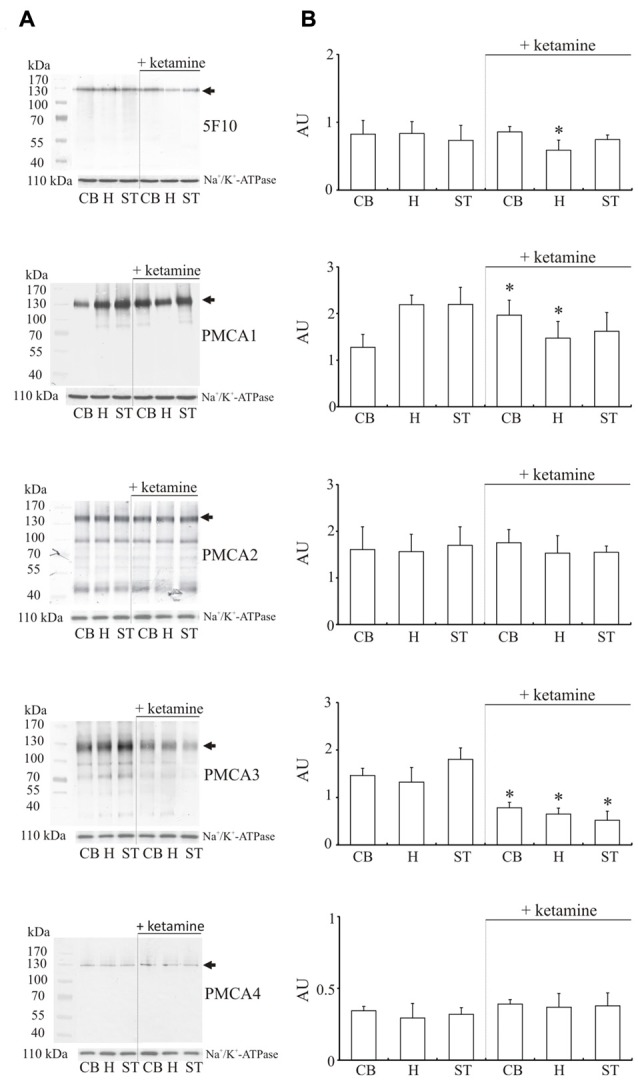
Western blot analysis of PMCA protein in synaptosomal membranes. The protein level was determined by immunoblotting **(A)** using 5F10 antibody recognizing all PMCA isoforms as well as isoform-specific antibodies. The intensity of bands was quantitated by densitometry **(B)**. The results are presented as AU obtained after normalization to endogenous Na^+^/K^+^-ATPase level. Representative blots are shown. Arrows indicate bands taken for quantitative analysis. *P < 0.05 ketamine treated vs. saline, *n* = 5. CB, cerebellum; H, hippocampus; ST, striatum.

Since total activity of PMCA depends on isoform composition in the membrane, in the next step we evaluated the effect of ketamine treatment on the amount of enzyme in examined brain regions (Figure 4). Single band of the predicted size (~130 kDa) was revealed for total PMCA, PMCA1 and PMCA4 but some lower molecular weight bands mainly at ~90 kDa and ~70 kDa corresponding probably to proteolytic degradation fragments were additionally detected for PMCA2 and PMCA3. The most prominent finding was a significant decrease of PMCA3 immunoreactivity in cerebellum, hippocampus and striatum (by 45 ± 9%, *p* < 0.001, Student’s *t*-test; by 50 ± 3%, *P* < 0.001, Student’s *t*-test and by 69 ± 11%, *P* < 0.001, Student’s *t*-test, respectively) isolated from ketamine-treated subjects. Similarly, there was a modest reduction in the level of PMCA1 (−32 ± 10%, *P* < 0.001, Student’s *t*-test), which was, however, detectable only in the hippocampal region. Conversely, the immunoreactivity of PMCA1 in cerebellum of ketamine-treated animals was significantly higher (+73 ± 22%, *P* < 0.01, Student’s *t*-test) than that from control group. No significant changes for PMCA2 and PMCA4 were detected in all three examined brain regions but statistically important PMCA2 decrease and adaptive up-regulation of PMCA4 were previously observed by us in cortical synaptosomes (Boczek et al., 2015b). Immunoprobing with 5F10 antibodies showed lowered total amount of enzyme (−30 ± 8%, *P* = 0.043, Student’s *t-test*) only in ketamine-treated hippocampal membranes, hinting at the existence of putative region-specific compensatory changes between PMCA isoforms, as was previously suggested for cortex (Boczek et al., 2015b). No increase in proteolytic degradation of PMCA2 was found and the weaker intensity of lower molecular bands of PMCA3 might reflect the overall decrease in PMCA3 level following ketamine administration rather than a change in pump proteolysis pattern of this transporter.

### Immunoblotting of NMDAR1, NMDAR2A, NMDAR2B and PSD95

**Figure 5:**
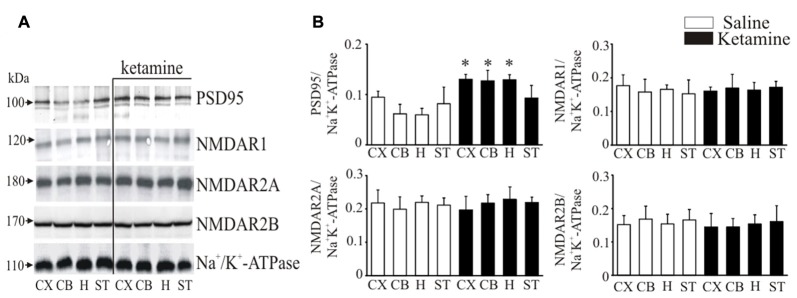
Western blot analysis of PSD95 and N-methyl-D-aspartic acid receptor (NMDAR) subunit protein level in synaptosomal membranes. The protein level of PSD95, NMDAR1, NMDAR2A and NMDAR2B was determined by immunoblotting **(A)** and the bands intensity corresponding to the target protein was densitometrically analyzed **(B)**. The results are expressed as AU obtained following normalization to endogenous Na^+^/K^+^-ATPase level. Representative blots are shown. *P < 0.05 ketamine treated vs. saline, *n* = 5. CX, cortex; CB, cerebellum; H, hippocampus; ST, striatum.

It is well documented that PMCA isoforms containing PDZ domain binding motif are dynamically regulated by PSD95, which is also essential for clustering and anchoring NMDA receptors at synapses (Garside et al., 2009). Thereby, we next determined ketamine effects on PSD95 and NMDA receptor subunit protein level in synaptosomal membranes (Figure 5). Immunoreactive protein bands corresponding to molecular masses of ~100 kDa and ~120 kDa were revealed for PSD95 and NMDAR1 subunit, respectively, and ~170 kDa for NMDAR2A or NMDAR2B subunits. The immunoreactivity of PSD95 in ketamine-treated rats was significantly higher in cortex (+35 ± 9%, *P* = 0.038, Student’s *t*-test), cerebellum (+78 ± 14%, *P* < 0.001, Student’s *t*-test) and hippocampus (+115 ± 14%, *P* < 0.001, Student’s *t*-test) than in control group. We did not detect any changes in the expression of NMDA receptor subunits between the two groups.

### Ketamine Effect on PSD95/NMDAR and PSD95/PMCA Interaction

**Figure 6:**
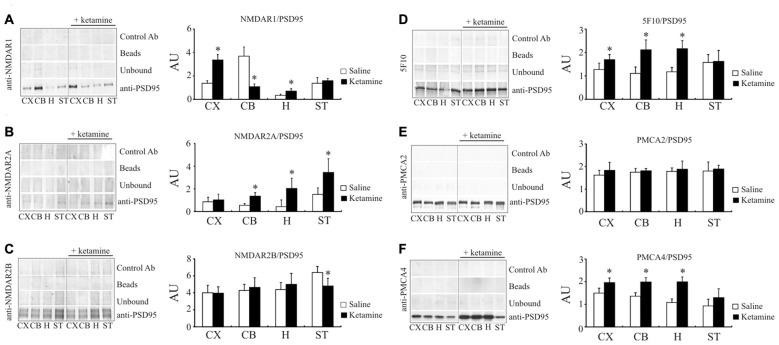
Interaction of PSD95 with NMDAR subunits and PMCA isoforms. Brain lysates were incubated with anti-PSD95 antibodies conjugated to sepharose beads. Immunocomplexes were recovered and resolved by SDS-PAGE and the membranes were next probed with anti-NMDAR1 **(A)**, anti-NMDAR2A **(B)**, anti-NMDAR2B **(C)**, 5F10 **(D)**, anti-PMCA2 **(E)** or anti-PMCA4 **(F)** antibodies. Negative controls included sepharose-linked secondary antibodies (Control Ab) or sepharose beads only. PSD95 was also immunoprobed in beads-unbound fraction (unbound). Representative blots are shown. The bands intensity were densitometically quantified and the results were expressed as AU defined as the optical density per mg of protein (OD/mg protein). *P < 0.05 ketamine treated vs. saline, *n* = 5. CX, cortex; CB, cerebellum; H, hippocampus; ST, striatum.

Following the study of Hahn et al. (2006) showing increased amounts of NMDAR1 and NMDAR2A subunits associated with PSD95 protein in individuals with schizophrenia, we next determined whether this effect can be reproduced by ketamine treatment. Indeed, the amount of NMDAR1 co-immunoprecipitated with PSD95 in cortical region was 2.45 ± 0.33 × higher (*P* < 0.01, Student’s *t*-test) in ketamine-treated group (Figure 6A). Similarly, higher level of PSD95/NMDAR1 complex was detected in hippocampus, but a 76 ± 7% decrease (*P* < 0.01, Man-Whitney U test) was observed in cerebellum. In contrast to the cortical region, where no changes for PSD95/NMDAR2A complex were found (Figure 6B), a significant increase in NMDAR2A immunoreactivity following PSD95 immunoprecipitation was observed in cerebellum (2.48 ± 0.56 ×, *P* < 0.001, Student’s *t*-test), hippocampus (4.83 ± 2.16 ×, *P* = 0.011, Mann-Whitney U test), and striatum (2.28 ± 0.8 ×, *P* = 0.012, Mann-Whitney U test). Slight, but statistically significant decrease in NMDAR2B amount co-immunoprecipitated with PSD95 (−23 ± 12%, *P* < 0.01, Mann-Whitney U test) was seen in striatal preparations from ketamine-treated animals (Figure 6C).

We next resolved whether ketamine could also modulate PMCA/PSD95 interaction. Ketamine treatment increased the amount of PMCAs immunoprecipitated with anti-PSD95 antibody in cortical (+33 ± 13%, *P* = 0.035, Student’s *t*-test), cerebellar (+95 ± 22%, *P* < 0.01, Student’s *t*-test) and hippocampal samples (+84 ± 25%, *P* < 0.01, Student’s *t*-test), but not in striatal preparations (Figure 6D). Further analysis with PMCA isoform-specific antibodies showed an ability of PMCA2 and PMCA4 to bind PSD95 (Figures 6E,F). No changes in PMCA2 quantity following PSD95 immunoprecipitation was detected in any of analyzed brain regions. However, increased PSD95/PMCA4 complex formation was demonstrated in cortex (+33 ± 7%, *P* = 0.15, Mann-Whitney U test), cerebellum (+48 ± 11%, *P* < 0.001, Student’s *t*-test) and hippocampus (+91 ± 14%, *P* < 0.001, Student’s *t-test*).

**Figure 7:**
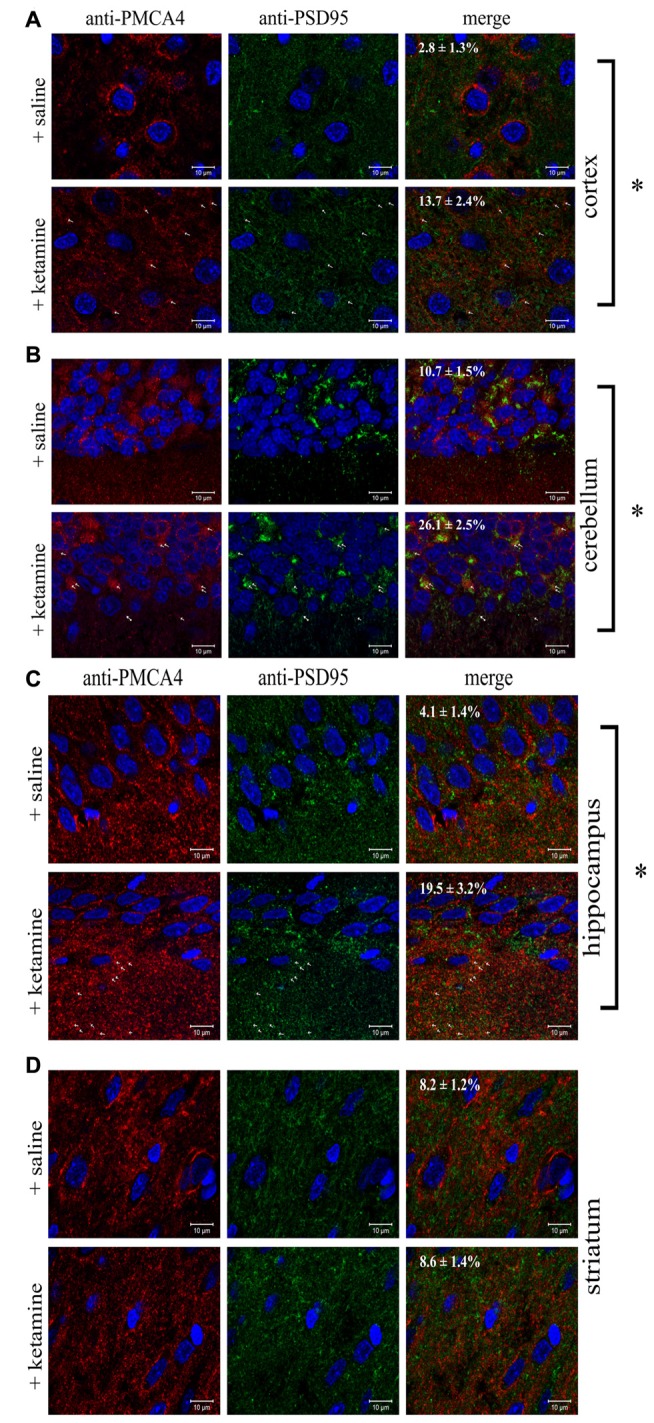
Colocalization of PSD95 and PMCA4 in brain regions. Sections of control or ketamine treated brain regions: cortex **(A)**, cerebellum **(B)**, hippocampus **(C)** and striatum **(D)** were prepared as described in “Materials and Methods” Section and immunostained with anti-PMCA4 primary and respective Alexa Fluor 568 conjugated secondary antibody (shown in red) as well as with anti-PSD95 primary antibody and respective Alexa Fluor 488 conjugated secondary antibody (shown in green). Cell nuclei were counterstained with Hoechst33342 and are shown in blue. Representative and corresponding regions of interest are shown for each brain section. Colocalization percentage (fraction of PMCA4-positive pixels which are also positive for PSD95) was calculated for several regions of interest and its mean value ± SD is presented on the microphotographs. The examples of overlapping pixels are indicated by arrows. Scale bars: 10 μM. *P < 0.0001 ketamine treated vs. saline, *n* = 9.

Since altered PMCA4/PSD95 interaction has not yet been reported in either psychotic subjects or upon-ketamine treatment, we asked whether the effect demonstrated by immunoprecipitation could be also observed directly by immunofluorescence in rat brain slices. Cell bodies were positive for both PMCA4 and PSD95 but, in contrast to PMCA, the fluorescence of PSD95 was dispersed in the cytosol but not in the plasma membrane (Figure 7). The double labeling of PMCA4 and PSD95 showed that punctuate PSD95 labelings were overlapped with PMCA4 labelings mostly in dendritic layers. However, the overlap between these two labelings was negligible in cell bodies. Confocal imaging also demonstrated ketamine-induced increase in colocalization between immunostained PMCA4 and PSD95 proteins, as assessed by changes in spatial overlap in PSD95-positive pixels with PMCA4 positive pixels in: cortex (*P* < 0.0001, Student’s *t*-test), cerebellum (*P* < 0.0001, Student’s *t*-test) and hippocampus (*P* < 0.0001, Student’s *t*-test).

### Ketamine Effect on Synaptosomal Glutamate Release

**Figure 8:**
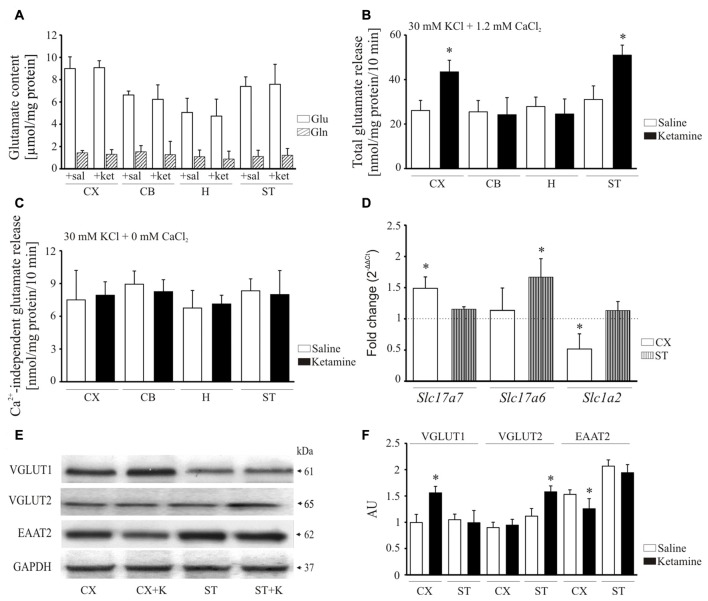
Effect of ketamine on synaptosomal glutamate concentration and release. Synaptosomal glutamate and glutamine content **(A)**. Total glutamate release evaluated by fluorometric assay over 10 min in the presence of 2 mM CaCl_2_ **(B)**. Ca^2+^-independent glutamate release measured in the absence of calcium in the reaction mixture **(C)**. The expression of *Slc17a7*, *Slc17a6* and *Slc1a2* evaluated using real-time PCR **(D)**. The fold change was calculated using comparative 2^∆∆Ct^ method following normalization to endogenous *Gapdh* expression used as an internal control. The expression level in saline-treated control was taken as 1 and is presented as a dotted line. The level of VGLUT1, VGLUT2 and excitatory amino acid transporters 2 (EAAT2) was analyzed by Western blot **(E)** and the intensity of bands was quantified densitometrically **(F)**. The results are presented as AU obtained after normalization to endogenous GAPDH level. *P < 0.05 ketamine treated vs. saline, *n* = 5. CX, cortex; CB, cerebellum; H, hippocampus; ST, striatum. CX + K; cortex + ketamine, ST + K, striatum + ketamine.

Psychotomimetic action of ketamine is thought to involve glutamate hyperactivity in the prefrontal cortex (Sleigh et al., 2014) but its effect on glutamate levels in other brain areas remains largely unknown. Therefore, in the final experiment we evaluated how ketamine might influence glutamate content and its release in cerebellum, hippocampus and striatum. A subanesthetic dose of the drug used here (30 mg/kg) was not associated with any changes in the total concentration of glutamate and glutamine (Figure 8A) suggesting that the drug does not affect glutamate/glutamine cycle.

Next, KCl-induced glutamate release from synaptosomal preparations was monitored by the increase in NADPH production in the presence of glutamate dehydrogenase and NADP^+^. Higher total glutamate release was observed from cortical (+66 ± 18%, *P* < 0.001, Student’s *t*-test) and striatal (+64 ± 14%, *P* < 0.001, Student’s *t*-test) synaptosomes isolated following ketamine challenge, but not in cerebellar and hippocampal preparations (Figure 8B). We then tested whether this increase reflected an effect on exocytotic vesicular release or on Ca^2+^- independent release attributable to reversal mode of glutamate transporter. The KCl-evoked glutamate secretion assessed in calcium-free conditions was shown to be less than 10 nmol/mg protein/10 min in all examined synaptosomal preparations and was unchanged by ketamine treatment (Figure 8C). This indicates that higher glutamate release observed in our study seems to be entirely associated with Ca^2+^-dependent exocytotic component.

To find out the cause of increased glutamate release, we made an attempt to investigate the effect of ketamine on vesicular (VGLUT1 and 2) and membrane (EAAT2) glutamate transporters (Figure 8D). The expression of *Slc17a7* (VGLUT1) was increased in cortex (+48 ± 12%, *P* < 0.01, Student’s *t*-test), whereas *Slc17a6* (VGLUT2) was induced in striatum (+66 ± 17%, *P* < 0.001, Student’s *t*-test). Conversely, the relative mRNA level of *Slc1a2* (EAAT2) was lower in cortex (−48 ± 17%, *P* < 0.01, Student’s *t*-test) of ketamine-treated animals. The level of VGLUT1 protein was higher by 36 ± 17% in cortex (*P* < 0.01, Student’s *t*-test) and VGLUT2 by 45 ± 10% in striatum (*P* < 0.01, Student’s *t*-test) following ketamine administration (Figures 8E,F). Similar to mRNA data, EAAT2 protein level was decreased in cortex (−30 ± 7%, *P* = 0.041, Student’s *t*-test) of ketamine-treated animals. Our data suggest that ketamine may increase glutamatergic neurotransmission by both promoting glutamate secretion from nerve endings and slowing down its clearance from extracellular space.

## Discussion

### Psychogenic Effect of Ketamine May be Associated with Dysregulated Cortico-Striatal Activity

One of the most observable effect of ketamine administration is a psychotic-like behavior in both humans (Krystal et al., 1994) and animal models (Lipska and Weinberger, 2000). Although the mechanism of psychogenic action of ketamine is not entirely clear, the established hypothesis postulates drug-mediated inhibition of NMDARs on GABAergic interneurons that disinhibit pyramidal neurons in prefrontal cortex (Homayoun and Moghaddam, 2007). In this study, we showed that repetitive injections of subanesthetic doses of ketamine (30 mg/kg) in rats produced immediate motor impairments including hyperlocomotion and stereotypies. Blockage of NMDA receptor by ketamine has been shown to mimic mostly negative, rather than positive symptoms, of psychotic behavior (Krystal et al., 2005; Stone et al., 2008). On the basis of clinical findings it is also thought that the expression of these symptoms is mediated by disruption of cortical glutamatergic neurotransmission and dysfunction in afferents and efferents of the nucleus accumbens, the main part of the ventral striatum (Javitt and Zukin, 1991; Olney and Farber, 1995). Indeed, previous studies have indicated that NMDA receptor antagonists produced hyperlocomotion and stereotypies by potentiating glutamatergic neurotransmission and the agents that inhibited glutamate release were able to reverse these behavioral changes (Deakin et al., 2008; Doyle et al., 2013). Moreover, the study of Takahata and Moghaddam (2003) suggests that NMDAR blockage-mediated motor effects were mediated though glutamatergic projections from the prefrontal cortex to the striatum. Therefore, disruption of cortico-striatal pathways seems to be involved in generation of hyperlocomotion and stereotypies induced by ketamine.

### Inhibition of PMCA Is Involved in Ketamine-Mediated Dysregulation of Ca^2+^ Homeostasis

Disruption of functional network connectivity and neurobehavioral homeostasis has been postulated as critical factors underlying generation of psychotic symptoms. The association of ketamine action with potential modifications of Ca^2+^ signaling/homeostasis is now brought to the forefront of ideas of molecular etiology of drug-induced psychotic states. Indeed, the overall effect of NMDAR blockage by ketamine is increased Ca^2+^ concentration in large population of neurons due to massive Ca^2+^ influx through non-NMDA glutamate gated ion channels including AMPA and kainate receptors (Lidow, 2003). Our previous study demonstrated significantly higher [Ca^2+^]_c_ in brain cells isolated from cortex, cerebellum, hippocampus and striatum following ketamine challenge (Lisek et al., 2016). Increase in [Ca^2+^]_c_ is thought to immediately stimulate PMCA, which is the first line of defense due to its Ca^2+^ affinity close to the resting Ca^2+^ concentration in neurons (Brini et al., 2017). We previously reported that ketamine might directly interfere with PMCA by interacting with two putative sites (Boczek et al., 2015b). Although this effect was initially observed for cortical pump, we show here that similar inhibitory potency of ketamine toward PMCA could be detected in other brain parts including cerebellum, hippocampus and striatum. This indicates that ketamine-induced PMCA inhibition may represent a general mechanism, irrespective of PMCA isoform or brain region, by which ketamine may affect Ca^2+^ concentration in neurons. The putative effect of such inhibition would be markedly reduced cellular Ca^2+^ clearance potency ultimately leading to calcium homeostasis deregulation.

Based on our data it is plausible that chronic ketamine treatment may activate compensatory up-regulation within PMCAs, especially PMCA1, which however does not seem to compensate for diminished total PMCA activity. We hypothesize that it may rather reflect potential changes in downstream signaling pathways involving PMCA. In this study, one of the most intriguing observations was ketamine-induced decrease in PMCA3 protein level in synaptosomal membranes isolated from cerebellum, hippocampus and striatum. Although very little data is available for this isoform, the *in vitro* studies suggested its functional interplay with P/Q-type voltage-dependent calcium channels (VDCCs) in the process of neurotransmitter release (Boczek et al., 2012). Overall, the extent of changes in PMCA protein upon ketamine treatment suggests that the level of expression cannot be entirely responsible for low PMCA activity. Instead, we assume that direct inhibitory effect of ketamine or structural modifications of pump protein by e.g., reactive oxygen species (ROS) may underlie the loss of catalytic activity. It has been shown that ketamine induces persistent increase in brain superoxide due to activation of NADPH oxidase (Behrens et al., 2007) and PMCA is highly vulnerable to ROS overload (Zaidi and Michaelis, 1999).

### Ketamine Interferes with the Formation of NMDAR/PSD95/PMCA4 Complexes at the Synaptic Membrane

PMCA is integrated into signaling pathways via multiple protein-protein interactions bringing the pump to “calcium signalosomes” together with ketamine-targeted NMDA receptors (DeMarco and Strehler, 2001; Kruger et al., 2010). Although the functions of these signaling centers have not been studied in details, based on our data it is tempting to speculate that NDMAR hypofunction may have additional effects on PMCA located in these multimeric complexes. Of paramount importance for PMCA and NMDAR scaffolding is PSD95 protein. However, the data derived from ketamine models showed confounding and often contradictory results with respect to PSD95 expression. In our study we found higher PSD95 protein level in cerebellum, hippocampus and striatum, but no changes in NMDAR subunit expression what does not yet exclude abnormal NMDAR expression and/or function. In line with PSD95 changes we also detected regionally specific alterations in PSD95/NMDAR interaction. PSD95 plays a crucial role the trafficking, membrane targeting and internalization of NDMAR (Elias et al., 2008; Feng and Zhang, 2009). Based on that, higher amount of NMDAR immunoprecipitated with PSD95 revealed in our study indicates higher functional coupling of both proteins *in vivo*. This, in turn, may reflect intensified targeting of NMDAR to the plasma membrane putatively aimed to restore reduced NMDAR-driven signaling. Moreover, intensified NMDAR1/PSD95 and PSD95/PMCA4 interactions in cortical region may indicate an assembly of local NMDAR1/PSD95/PMCA4 densities. Formation of such structures could, at least partially, counterbalance higher Ca^2+^ influx through e.g., AMPA receptors due to PMCA4 targeting in a close proximity to Ca^2+^ entryways. It has been recently suggested that PSD95-induced clustering of PMCA4 is highly effective (Padányi et al., 2009) and PSD95 facilitates raft association of this isoform (Sepúlveda et al., 2006). Increased concentration of PMCA4 can, in turn, induce its oligomerization leading to stimulation of pump activity (Vorherr et al., 1991; Kosk-Kosicka et al., 1994). Therefore, PSD95-mediated formation of PMCA4 clusters could significantly improve calcium extrusion from cortex, cerebellum and hippocampus. Contrary, lack of enhanced PSD95/PMCA4 interaction in striatum may shed some light on why the resting [Ca^2+^]_c_ observed in our previous study, was the highest in this brain region. As the formation of oligomers involves CaM-binding domain (Vorherr et al., 1991; Kosk-Kosicka et al., 1994), it is plausible that reduced CaM stimulation could result not only from direct inhibition of PMCA, but also from decreased accessibility of CaM-binding domain due to formation of oligomers. Overall, as both NMDA and AMPA receptor but also PMCA4 are located to lipid rafts, this may suggest that PMCA4 could play a predominant role in Ca^2+^ clearance following ketamine-induced but non-NMDAR mediated calcium entry.

Interestingly, higher amounts of PSD95/NMDAR2A were observed in cerebellum, hippocampus and striatum. It has been demonstrated that interaction between PSD95 and NMDAR2A controls the synaptic development and the amount of PSD95/NMDAR2A complex indicates maturation of NMDAR-containing synapses (Bard and Groc, 2011). Then, our results could indicate an enhanced NMDAR2A insertion at the synapse. Synaptic NMDAR-dependent activation of ERK1/2 contributes to CREB activation, which is a key component in Ca^2+^-dependent pro-survival pathway. Ketamine was shown to dramatically decrease ERK1/2 activation lasting for at least 24 h (Straiko et al., 2009) what is thought to contribute to cell death. The prefrontal cortex is especially vulnerable to ketamine and several *in vivo* and *in vitro* studies (Takadera et al., 2006; Sun et al., 2014; Zhao et al., 2016) reported apoptosis of cortical neurons in response to drug treatment. In light of these findings, increased PSD95/NMDAR2A clustering could have a beneficial effect on neuronal viability.

### NMDAR/PSD95/PMCA4 Complexes as Mediators of Presynaptic Glutamate Release

The possibility exists that PMCA, PSD95 and NMDAR are located within a ternary postsynaptic signaling complex, but the functional relation of this complex to presynaptic glutamate release has not been shown so far. It is known that postsynaptic NMDAR could regulate presynaptic function via retrograde trans-synaptic signal sent from the postsynaptic dendrite to its presynaptic partner (Fitzsimonds and Poo, 1998). In the standard sequence of events, presynaptically released glutamate binds to both AMPA and NMDA receptors but AMPA-driven depolarization is necessary to release Mg^2+^ blockage of NMDAR allowing for Ca^2+^ influx. Blockage of NMDAR pore by ketamine switches off NMDAR-downstream signaling but leads to excessive Ca^2+^-influx via other glutamate-gated channels, as was discussed above. Coupling PMCA to NMDAR/PSD95 complexes located close to these Ca^2+^ entry sites could potentially counterbalance Ca^2+^ overflow; however, ketamine-mediated loss of PMCA activity makes this system relatively ineffective. PMCA inhibition may thus lead to the generation of an abnormally prolonged Ca^2+^ signal and over-activation of retrograde messenger(s) synthesis, ultimately producing long-lasting increase in glutamate synthesis and release from presynaptic terminal. One of the strongest candidate for retrograde messenger is NO, which is released in Ca^2+^-dependent manner (Garthwaite et al., 1988). It has been shown that NO is synthesized in postsynaptic cells and enhances neurotransmitter release in NMDA-independent fashion when applied exogenously (Bon et al., 1992; Zhuo et al., 1993). The activity of neuronal nitric oxide synthase (nNOS) can be regulated by PSD95/NMDAR2B complex (Lai et al., 2011) and ketamine may promote nNOS/NMDAR2B interaction via PSD95 (Lecointre et al., 2015). PMCA binds nNOS as well, but this interaction is thought to slow down NO generation (Duan et al., 2013). Despite this, high [Ca^2+^]_c_ observed by us upon ketamine treatment may increase nNOS activity via a Ca^2+^/CaM-dependent mechanism (Förstermann and Sessa, 2012), while PMCA inhibition by ketamine may additionally potentiate NO production. Hence, the association of signaling proteins such as nNOS with NMDAR2B/PSD95 at the postsynaptic membrane can affect retrograde messenger(s) production and thus increase presynaptic glutamate release. Postsynaptic complexes with PSD95 have been shown to affect retrograde modulation of the release probability and to determine presynaptic neurotransmission (Futai et al., 2007).

### Ketamine Effect on Glutamate Secretion and Re-Uptake

Our data on ketamine-induced increase in glutamate release from cortical and striatal synaptosomes are consistent with the reports of others (Moghaddam et al., 1997; Chowdhury et al., 2012), suggesting that this phenomenon may be specific only to subchronic doses of the drug. The fast secretion of glutamate requires its transport from the cytosol into secretory vesicles and is mediated by VGLUTs. In our study, higher expression and protein content of VGLUT1 and VGLUT2 were found in cortex and striatum, respectively. VGLUT1-expressing neurons are found predominantly in hippocampus, thalamus as well as in cerebral and cerebellar cortices (Ni et al., 1995; Bellocchio et al., 1998; Herzog et al., 2001) while VGLUT2 is abundant in subcortical excitatory neurons (Ni et al., 1995; Fremeau et al., 2001; Herzog et al., 2001). Although, striatal neurons do not directly express VGLUT1 and VGLUT2, striatum receives two glutamatergic afferents: one from VGLUT1-positive cortico-striatal pathway and the other form VGLUT2-postitive thalamo-striatal pathway (Smith and Bolam, 1990; Kaneko and Fujiyama, 2002; Smith et al., 2004). Indeed, a study on a single-labeled tissue demonstrated striatal enrichment in the terminals immunolabeled for either VGLUT1 or VGLUT2 (Lei et al., 2013). Therefore, increased amount of VGLUT1 mRNA and protein level observed in this study may reflect an authentic induction of gene expression in cortical neurons whereas higher VGLUT2 expression can be attributable to changes in afferents possibly form thalamic or other subcortical regions. Our results suggest that increased VGLUT1-2 may contribute to abnormal glutamate secretion, given that VGLUT expression has been shown to directly influence quantal glutamate release (Wilson et al., 2005). It has also been reported that PMCAs colocalize with VGLUT1 in presynaptic terminals (Jensen et al., 2007), raising the possibility that PMCA could ensure a tight coupling between local Ca^2+^ efflux and neurotransmitter release. In line with that, removal of PMCA function enhanced glutamate release (Garside et al., 2009) and elevated residual calcium within the presynaptic terminal (Jensen et al., 2007). Therefore, strong sensitivity of PMCA to ketamine inhibition coinciding with higher neuronal [Ca^2+^]_c_ may profoundly contribute to altered glutamate secretion in our experimental model. This effect may not, however, be limited only to the interaction with VGLUTs but may also involve PMCA-dependent generation of Ca^2+^ rises within the active zones, the sites of neurotransmitter release. PMCA is clustered within these specialized calcium microdomains (Blaustein et al., 2002), so a disruption in PMCA-mediated presynaptic Ca^2+^ clearance by ketamine can trigger local Ca^2+^ rises facilitating vesicular fusion and secretion of glutamate. This creates another potential pathway by which PMCA can mediate Ca^2+^-dependent neurotransmission defects observed in psychotic states.

Abnormal secretion of glutamate does not always reflect enhanced glutamatergic neurotransmission, as the excess of this neurotransmitter in the synaptic cleft can be efficiently taken up by the system of excitatory amino acid transporters (EAATs). EAAT2 is the principal mechanism of glutamate clearance from the synapse (Danbolt, 2001). In this study, we detected downregulation of EAAT2 in the cortical regions following ketamine challenge, consistent with a previous report showing long-lasting decrease in EAAT2 alongside alterations in EEG and memory in chronically treated animals (Featherstone et al., 2012). Giving that EAAT2 is responsible for over 90% of glutamate uptake in adult brain (Kanai and Hediger, 2003), any changes in the amount of this transporter would lead to glutamate accumulation in the synaptic cleft and over-activation of glutamate receptors. The excess of glutamate may also activate other extra-synaptic targets leading to propagation of aberrant signaling. Interestingly, the changes in EAAT2 and VGLUT1 level seem to be inversely correlated in our study, suggesting the existence of a functional relationship between both transporters already reported in certain neurological deficits (Sánchez-Mendoza et al., 2010). It has been demonstrated that repeated ketamine administration increased the number of glial fibrillary acidic protein (GFAP)-positive astrocytes but led to the cortical neuron damage (Liu et al., 2011). Therefore, differential expression of EAAT2 and VGLUT1 can be attributed to ketamine-induced activation of different signaling pathways rather than changes in the relative size of neuronal and glial populations. One proposed mechanism involves ketamine-mediated reduction of PP2A and PI3K-dependent Akt phosphorylation. This, in turn, is assumed to decrease NF-kB phosphorylation, ultimately driving EAAT2 downregulation (Li et al., 2006). Interestingly, no such regulation was found for VGLUTs. Taken together, the reduction in EAAT2, which seems to go against the homeostatic role of VGLUTs, can be driven by different signaling pathways and might reflect a compensatory, yet pathogenic mechanism aimed to increase glutamate level in the synaptic cleft, in a futile attempt to restore defective NMDAR function. As was reported in the study of functional EAAT2 knockdown (Rothstein et al., 1996), it is plausible that EAAT2 downregulation by ketamine observed in our study may eventually increase glutamate concentration to neurotoxic values, leading to excitotoxic neurodegeneration.

## Conclusion

**Figure 9:**
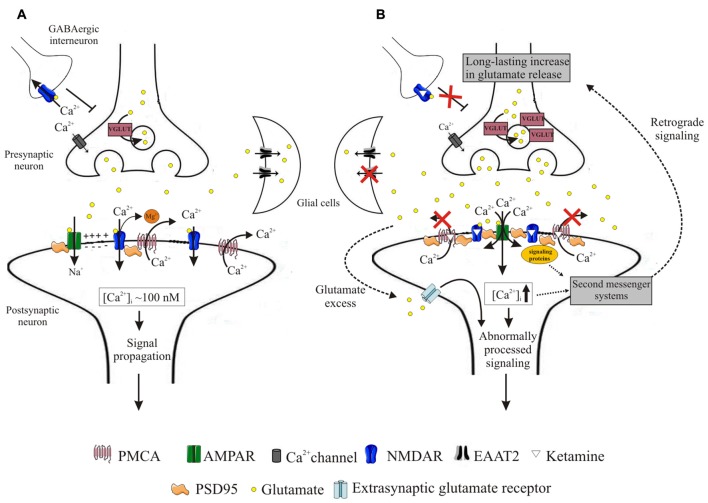
Schematic representation of events induced by ketamine. During neurotransmission, presynaptically released glutamate binds to both AMPA and NMDA receptors. Activation of AMPA receptors depolarizes postsynaptic membrane, driving Mg^2+^ out of the NMDA receptor pore. This allows for Ca^2+^ entry to the cell, from whence it is subsequently rapidly extruded into the synaptic cleft by high-affinity PMCA. This allows for tight control of intrasynaptic [Ca^2+^]_c_ rises and propagation of unaltered downstream signals **(A)**. Blockage of NMDA receptors by ketamine induces massive Ca^2+^ influx through AMPA receptors. Ketamine also inhibits PMCA, depleting cellular Ca^2+^-clearing potency and leading to elevated [Ca^2+^]_c_ within the synapse. This triggers formation of NMDAR/PSD95/PMCA complexes, putatively located close to the Ca^2+^ entry sites and acting to restore resting [Ca^2+^]_c_. These complexes may further recruit signaling molecules but transmission of aberrant retrograde signal leads to a long-lasting increase in presynaptic glutamate secretion facilitated by overexpression of VGLUTs. Once released, glutamate is not effectively cleared due to downregulation of glial EAAT2 in what seems to constitute another adaptive change to restore defective postsynaptic NMDA receptor-mediated signaling. The excess of glutamate in the synaptic cleft may activate extra-synaptic receptors and together with dysregulated Ca^2+^ homeostasis contribute to the propagation of abnormal signals **(B)**.

In conclusion, our data demonstrate that inhibitory action of ketamine on PMCA is a general phenomenon, not limited only to cortical structures. Although the effect of PMCA blockage seems to globally contribute to Ca^2+^ homeostasis dysregulation, region-specific alterations in PSD95 clustering with NMDA receptor subunits and/or PMCA4 may shape local calcium signaling and modulate cell response to ketamine administration. As shown in schematic Figure 9, incorrectly progressing postsynaptic Ca^2+^ signal, mainly due to ketamine interference with Ca^2+^ clearance, may induce a feedback response leading to long-lasting increases in presynaptic glutamate secretion. The aberrant glutamatergic neurotransmission may in turn produce aberrant signal leading to generation of psychotic-like effects and potential excitotoxicity. Our work points out a novel mechanism by which ketamine-triggered postsynaptic changes may affect presynaptic neurotransmitter release and indicates molecular components of neurosignaling involved in psychomimetic action of ketamine.

## Author Contributions

ML, BF, MS, LP, LZ, FG and TB: participated in research design; wrote or contributed to the writing of the manuscript. ML, BF, MS and TB: conducted the experiments. ML, MS, LP, FG, LZ and TB: performed data analysis.

## Conflict of Interest Statement

The authors declare that the research was conducted in the absence of any commercial or financial relationships that could be construed as a potential conflict of interest.
